# Cognitive decline in Huntington’s disease in the Digitalized Arithmetic Task (DAT)

**DOI:** 10.1371/journal.pone.0253064

**Published:** 2021-08-23

**Authors:** Marine Lunven, Jennifer Hamet Bagnou, Katia Youssov, Alexis Gabadinho, Rafika Fliss, Justine Montillot, Etienne Audureau, Blanche Bapst, Graça Morgado, Ralf Reilmann, Robin Schubert, Monica Busse, David Craufurd, Renaud Massart, Anne Rosser, Anne-Catherine Bachoud-Lévi

**Affiliations:** 1 Département d’Etudes Cognitives, École normale supérieure, PSL University, Paris, France; 2 University Paris Est Creteil, INSERM U955, Institut Mondor de Recherche Biomédicale, Equipe NeuroPsychologie Interventionnelle, Creteil, France; 3 AP-HP, Hôpital Henri Mondor-Albert Chenevier, Centre de référence Maladie de Huntington, Service de Neurologie, Créteil, France; 4 NeurATRIS, Créteil, France; 5 Clinical Epidemiology and Ageing, Service de santé publique, Henri Mondor Hospital, AP-HP, Créteil, France; 6 Department of Neuroradiology, AP-HP, Henri Mondor University Hospital, Créteil, France; 7 Faculty of Medicine, Université Paris Est Créteil, Créteil, France; 8 Centre d’Investigation Clinique, Hôpital Henri Mondor, Créteil, France; 9 George-Huntington-Institute, Technology-Park, Muenster, Germany; 10 Department of Clinical Radiology University of Muenster, Muenster, Germany; 11 Dept. of Neurodegeneration and Hertie Institute for Clinical Brain Research, University of Tuebingen, Tuebingen, Germany; 12 Centre for Trials Research, Cardiff University, United Kingdom; 13 NMHRI, School of Medicine, and Brain Repair Group, School of Biosciences, Cardiff University, Cardiff, United Kingdom; 14 Manchester Centre for Genomic Medicine, St Mary’s Hospital, Manchester University NHS Foundation Trust, Manchester Academic Health Science Centre, Manchester, United Kingdom; 15 Division of Evolution and Genomic Sciences, School of Biological Sciences, Faculty of Biology, Medicine and Health, University of Manchester, Manchester Academic Health Science Centre, Manchester, United Kingdom; 16 Wales Brain Research And Intracranial Neurotherapeutics (BRAIN) unit, Wales; Istituto Di Ricerche Farmacologiche Mario Negri, ITALY

## Abstract

**Background:**

Efficient cognitive tasks sensitive to longitudinal deterioration in small cohorts of Huntington’s disease (HD) patients are lacking in HD research. We thus developed and assessed the digitized arithmetic task (DAT), which combines inner language and executive functions in approximately 4 minutes.

**Methods:**

We assessed the psychometric properties of DAT in three languages, across four European sites, in 77 early-stage HD patients (age: 52 ± 11 years; 27 females), and 57 controls (age: 50 ± 10, 31 females). Forty-eight HD patients and 34 controls were followed up to one year with 96 participants who underwent MRI brain imaging (HD patients = 46) at baseline and 50 participants (HD patients = 22) at one year. Linear mixed models and Pearson correlations were used to assess associations with clinical assessment.

**Results:**

At baseline, HD patients were less accurate (p = 0.0002) with increased response time (p<0.0001) when compared to DAT in controls. Test-retest reliability in HD patients ranged from good to excellent for response time (range: 0.63–0.79) and from questionable to acceptable for accuracy (range: r = 0.52–0.69). Only DAT, the Mattis Dementia Rating Scale, the Symbol Digit Modalities Test, and Total Functional Capacity scores were able to detect a decline within a one-year follow-up in HD patients (all p< 0.05). In contrast with all the other cognitive tasks, DAT correlated with striatal atrophy over time (p = 0.037) but not with motor impairment.

**Conclusions:**

DAT is fast, reliable, motor-free, applicable in several languages, and able to unmask cognitive decline correlated with striatal atrophy in small cohorts of HD patients. This likely makes it a useful endpoint in future trials for HD and other neurodegenerative diseases.

## Introduction

Huntington’s disease (HD) is an inherited, autosomal, neurodegenerative disorder caused by a CAG repeat extension in the huntingtin gene on chromosome 4 [1]. Thanks to a better understanding of the condition, promising gene therapies appear to be on the horizon [2,3]. Although cognitive and behavioral symptoms are the most detrimental for patients and their families, clinical trials mainly focus on motor symptoms and general functional capacity because widely-endorsed cognitive endpoints are lacking [4]. Evaluating the risk/benefit of disease-modifying phase I and II trials requires validated brief-objective cognitive assessments sensitive to decline in relatively small cohorts of patients [5]. Because many trials are conducted across countries in different languages, cultural variation should not affect task dissemination and performance. Yet, most of the available tasks are either not validated longitudinally (e.g. the Montreal Cognitive Assessment [5,6]), or lack psychometric evaluations in HD (Mini-Mental State Examination test and of the Mattis Dementia Rating Scale [5]). Currently, most studies use the cognitive section of the Unified Huntington’s Disease Rating Scale (UHDRS) [7]. Despite Stroop Word reading, Stroop Color naming, and Symbol Digit Modalities Test usually capturing patients’ decline over 2 to 3 years in both small and large cohorts (N>50), Stroop Interference and Letter Fluency have a tendency to yield conflicting results without consistently showing decline [8–10]. Interestingly, tests assessing psycho-motor and executive capacities appear the most sensitive to disease progression, especially when these tests are time-dependent; this prompted the emergence of a new generation of digitized tasks, which allow for controlling presentation time of stimuli and recording a precise response time. Moreover, digitalized assessment has the advantage of improving standardization across sites and limiting potential investigator bias. For example, the promising Huntington’s Disease Cognitive Assessment Battery [11] concatenates six cognitive tasks selected for their ability to capture disease progression in previous HD longitudinal studies, half of which were digitalized. When comparing HD patients and controls, they found large effect sizes at baseline, an expected retest effect [12], and a stabilization of performance within 2 months. Results on a longer follow-up period are expected. In contrast, the Cambridge Neuropsychological Test Automated Battery (CANTAB) followed a small cohort of HD patients with neural transplants [13] and HD patients with mild to moderate impairment over three years [14] for over a decade. Out of the 19 battery subtests, two were able to detect a decline (the Tower London and the Set Shifting Task) after 6 years of follow-up. This battery was recently used in a cross-sectional study in which the authors did not find significant cognitive impairment in premanifest gene carriers far from onset when compared to healthy controls [15].

Executive function and language are commonly impaired in the early stages of HD [16–18] and are closely related to disease progression [9,14,19]. Combining these two functions should therefore increase the chances of observing a decline in longitudinal follow-up of relatively small cohorts of patients. However, it then appears necessary to resolve the limits of linguistic translation of language assessments. It has been shown that simple subtraction and multiplication tasks involve language-related brain networks [20]. The advantage of using arithmetic tasks is that they provide a window for both language processing and executive functions without the disadvantage drawback of translation limitation; multiplication is sensitive to language being learned through verbal code, while subtraction applies “carry-over” rules that require executive function [21]. In a previous cross-sectional study in early-stage HD patients [18,21] and premanifest gene carriers [22], we found that HD patients performed worse than healthy participants in both multiplication and subtraction paper and pencil tasks. We thus digitalized our arithmetic task (Teichmann et al. 2005) [21] as the Digitalized Arithmetic Task (DAT) to provide an objective cognitive assessment with minimal examiner interference. We included both the control of stimuli presentation and the response time recording (accuracy and response time). Then, we adapted it from French to both English and German. First, we validated and assessed DAT’s psychometric properties by using two subsequent baselines with a one-month interval to limit the impact of the retest effect and of any potential statistical noise from participants previously exposed to testing [12]. Second, we assessed DAT decline over eleven months by comparing performance between Month 1 and Month 12. We then measured the association between DAT performance and striatal atrophy in a subgroup of participants with available MRI scans.

## Methods

### Participants

Out of the 185 patients in the European observational longitudinal study (Repair-HD, http://www.repair-hd.eu), which aims to establish a new protocol for assessment of innovative therapies in Huntington’s disease, 77 HD patients and 57 healthy controls matched for age (F(1,132) = 0.91; P = 0.34), education level (F(1,131) = 0.21; P = 0.64) and handedness (X-squared = 0.75, df = 2, P = 0.68) were included in the present study (Table 1). There were more males in the HD group than in controls (X-squared = 4.22, df = 1, P = 0.039). Participants were recruited from 4 sites (Cardiff, UK; Créteil, France; Manchester, UK; and Muenster, Germany). The inclusion criteria for HD patients were (i) confirmed CAG expansion (≥ 38 CAG repeats) and (ii) presence of minimal to moderate clinical impairments at stages 1 or 2 of the disease according to the UHDRS Total Functional Capacity (TFC) scores (TFC ≥ 7) [23]. Matched healthy controls were spouses or partners of HD patients, gene-negative siblings, or persons not related to HD patients. Exclusion criteria included alcohol or substance abuse, and neurological co-morbidity. The present study obtained ethics approval from the local research ethics committee (CPP Ile de France III) and ethical approval was granted by the CAPIT-HD Beta study (NCT 03119246, https://clinicaltrials.gov/CAPIT-HD). Written informed consent was obtained from each participant.

**Table 1:** Participant demographics at baseline (M0).

	Controls	HD patients
Number	57	77
(Cardiff/Créteil/Manchester/Muenster)	(3/34/3/17)	(11/36/5/25)
Laterality	1A/51R/5L	2A/71R/4L
Sex	31F/26M	27F/50M
Age (years)	50.52 ± 10.06	52.31 ± 11.28
[range]	[26.17–70.01]	[23.21–72.98]
Education (years)	14.05 ± 3.23	14.30 ± 2.98^#^
[range]	[8–24]	[9–20]
TFC (M0)	13.00 ± 0.00	10.91 ± 1.44
[range]	[13–13]	[7–13]
TMS (M0)	0.63 ± 1.11	28.37 ± 14.79
[range]	[0–5]	[1–58]
CAG repeat	-	43.43 ± 3.73
[range]	-	[38–62]
Age of onset (years)	-	48.85 ± 10.63^##^
[range]	-	[21–69]
Disease duration (years)	-	4.58 ± 3.78^##^
[range]	-	[0.15–19.20]
Disease burden score	-	385.66 ± 98.22
[range]		[129.71–672.95]

F Female, M Male, R Right, L Left, A Ambidextrous, TFC Total Functional Capacity; disease burden score = age × (CAG length– 35.5) [24]

^#^1 missing data

^##^5 missing data.

Unless otherwise specified, values are means ± standard deviations.

### General assessment

Clinical variables assessing motor and cognitive abilities were selected across the UHDRS [7]. The motor measure used was the Total Motor Score (TMS), defined as the sum of all individual motor abnormality ratings (oculomotor, bradykinesia, rigidity, dystonia, and chorea), with a higher score indicating a more severe motor impairment. The functional outcome used was the Total Functional Capacity (TFC) score, a 5-item clinician rating scale assessing occupation, finances, domestic chores, activities of daily living, and level of care. TFC ranges from 0 to 13 with greater scores indicating higher functioning capacity. Cognitive measures included the Letter Verbal Fluency Task over one minute [25], the Symbol Digit Modalities Test (SDMT), and the Stroop tests (Colour, Word, and Interference). Additionally, participants performed the Mattis Dementia Rating Scale (MDRS) [26] and the Hopkins Verbal Learning Memory Test (HVLMT) [27].

### Digitalized arithmetic task (DAT)

We adapted the paper and pencil arithmetic task [21] into a computerized form. DAT contains a relatively small number of trials (N = 40) to maintain brevity for clinical practice. Twenty multiplication and 20 subtraction problems with their given results were matched on the number of digits they contained. In half of the cases, the proposed result was false; in the other half, it was correct. Participants were asked to indicate the correctness of each given result by clicking either “correct” (on the right) or “false” (on the left) on the screen using the mouse, with no time limit to respond. Response time (RT) and accuracy were recorded for each trial. Stimulus presentation and response recording were performed in Python, using the Psychopy toolbox (https://www.psychopy.org/), and the task was completed on different laptops (Cardiff: 1366 x 768; Créteil: 1440 x 900; Manchester: 1536 x 864 and Muenster: 1920 x 1080 pixels). The task lasted 2.59 ± 0.84 minutes in controls and 4.03 ± 1.51 minutes in HD patients.

We analyzed median response time (RT) of correct responses and accuracy (percentage of correct responses) for each subject at each time of evaluation. The median was chosen for RT because of its superior reliability when using a small number of items (N = 40). Here, we provide measures combining data in the entire DAT (Arithmetic RT and Arithmetic accuracy), results for multiplication and subtraction are displayed separately in S1 Table. We also analyzed the Arithmetic Inverse Efficiency Score (IES) [28], an index that accounts for both RT and accuracy. This score reduces the impact of the speed-accuracy trade-off [29], as the balance between the participant motivation to answer quickly or accurately. It is particularly useful in longitudinal studies where the stage of the disease may influence the patients’ response behaviour. The IES was computed by dividing median RT (in seconds) of correct responses by accuracy, with a higher IES indicating lower performance.

### MRI data acquisition and pre-processing

Brain MRI acquisition was performed at two centres. At Henri Mondor Hospital (Créteil, France), participants underwent a high-resolution brain MRI scan on a Siemens Skyra including T1 3D anatomical MP-RAGE images (repetition time: 2300 ms; echo time: 2900 ms; inversion time: 900 ms; flip angle: 9°; acquisition matrix: 256 x 240; slice thickness: 1.2 mm, no inter-slice gap, 176 sagittal sections). At the George Huntington Institute (Muenster, Germany), participants underwent a high-resolution brain MRI scan on a Philips Medical Systems including T1 3D anatomical MP-RAGE images (repetition time: 6770 ms; echo time: 3130 ms; inversion time: 900 ms; flip angle: 9°; acquisition matrix: 256 x 256; slice thickness: 1.2 mm, inter-slice gap: 1.2 mm, 170 sagittal sections).

We used FreeSurfer (https://surfer.nmr.mgh.harvard.edu/) [30] to calculate subcortical volumes both cross-sectionally and longitudinally (using a dedicated method implemented in FreeSurfer for longitudinal follow-up). The percentage of striatal volume relative to the estimated intracranial volume was obtained from the volumes of the caudate nucleus, ventral striatum, and putamen. There was no significant difference in striatal volumes in control participants between France and Germany (P>0.5).

### Procedure

**Fig 1:**
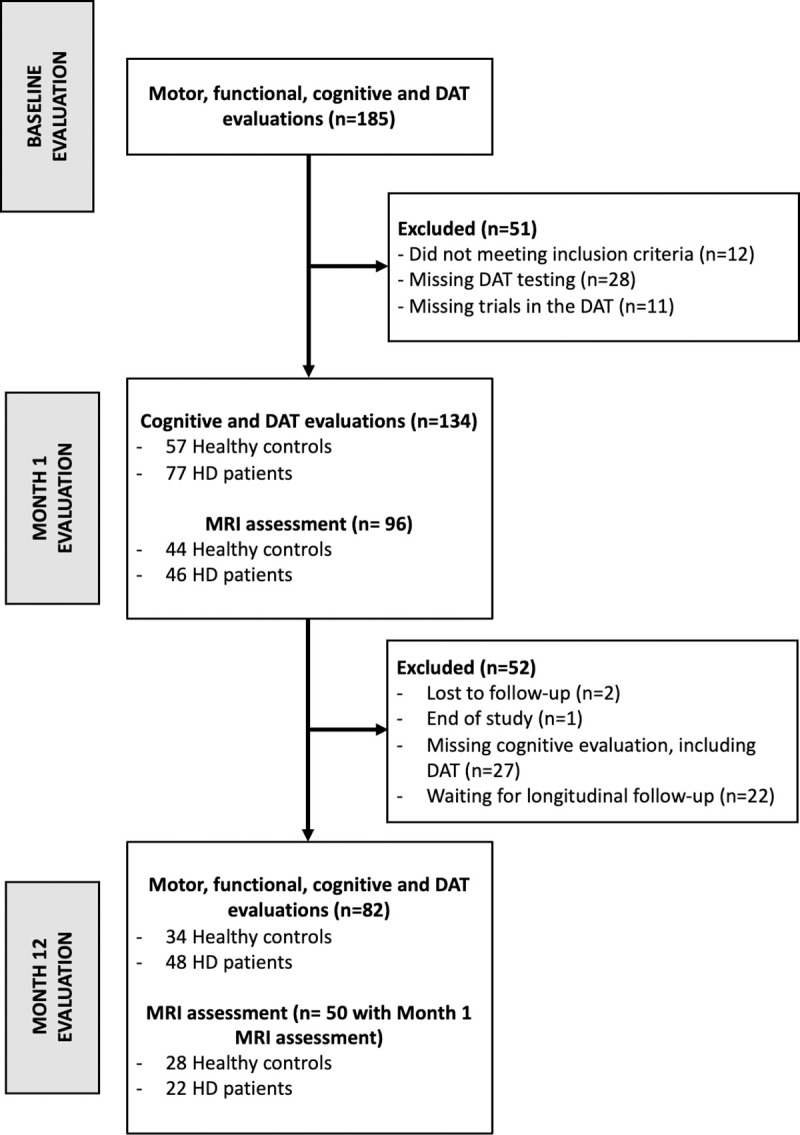
Repair-HD study participant disposition in this current study. DAT Digitalized Arithmetic Task, HD Huntington Disease.

The study flow chart and the demographic and clinical characteristics of participants at baseline are displayed in Fig 1 and Table 1, respectively. 134 Participants performed cognitive assessments (including DAT) at baseline (M0) and one month (M1) later. As shown in Fig 1, 12 subjects were excluded after initial screening as they were either controls with cognitive impairment identified by neuropsychological and neurological assessments or HD patients at Stage 3 of the disease. 28 participants across the four centers did not perform the cognitive computerized evaluation (including the DAT) due to a shortage of specialists for this assessment in some centers. We also excluded 11 participants from analysis due to technical issues during the test, with missing trials at the beginning of the study related to a default in digitalized task implementation. Eighty-two (48 HD patients and 34 healthy controls) were followed-up to M12. Follow-up could not be performed for 5 participants (2 lost to follow-up, 1 end of study). 27 participants could did not complete from the cognitive evaluation during the M12 follow-up due to a shortage of specialists for this assessment in some centers. Finally, longitudinal data for 22 participants remain pending.

The motor (TMS) and functional (TFC) evaluations were only proposed at M0 and M12. Brain MRI scans were obtained for 96 participants (46 HD patients) at M1 and 50 participants (22 HD patients) at M12. Clinical, motor, cognitive evaluations and brain imaging acquisition were carried out over two days.

### Statistical analysis

#### Clinical and cognitive metrics

Missing data in classic cognitive tests were imputed using the “missForest” package implemented in R using Random Forest.

Linear models were performed at M0 on cognitive and DAT metrics to assess performance differences between healthy controls and HD patients. Age, sex, education level, and TMS were added as covariates. Adding TMS as a covariate allowed for limiting the motor impact on cognitive performance. Pearson correlation coefficients were used to assess associations between DAT’s measures and cognitive assessments in HD patients at M0 and to assess the reliability of these measures between M0 and M1 evaluations in all participants.

We used longitudinal linear mixed models (M0-M1 or M1-M12 analysis) on motor, functional, DAT, and cognitive measures using the “lme4” and “lmerTest” packages in R software [31]. Main effects of fixed factors (and their respective interactions) were assessed by model comparisons (likelihood ratio tests). Participants and languages were added as random intercept factors with uncorrelated random intercepts and slopes within participant. Age, sex, education level, and TMS (only in cognitive analysis) were added as covariates. Post hoc analyses were completed using the “emmeans” package implemented in R software with Tukey’s correction method for multiple comparisons. In the longitudinal M1-M12 study, we computed repeated-measures Cohen’s f effect sizes for each cognitive test in HD patients using the statistical mixed-effects models results as implemented in the “effectsize” library.

#### Brain imaging analysis

At M1, linear regression was used to compare striatal volumes between groups. We also evaluated the association between cognitive or DAT measures and the striatal volume in HD patients using striatal volume as the predictor.

Least-squares linear regression was used to compare longitudinal M1-M12 change in striatal volume between groups. The association between longitudinal differences in motor, functional, DAT, and cognitive measures (delta M12 –M1) and longitudinal differences in striatal volume (delta M12 –M1) was evaluated in HD patients using linear regression with inclusion of time, striatal volume, and its interaction with time as predictors. All statistical analyses conducted with brain imaging data were adjusted for age, sex, education, and MRI centre.

## Results

### Baseline (M0) and Month 1 (M1) analysis

At M0, controls performed better than HD patients on each of the paper and pencil tasks (UHDRS cognitive assessments, MDRS, and HVLMT) (S2 Table, all Ps <0.05). In HD patients, TFC did not correlate with paper and pencil task performance, while significant negative correlations were found between TMS and executive performances (S2 Fig: SDMT, the three parts of the Stroop).

**Fig 2:**
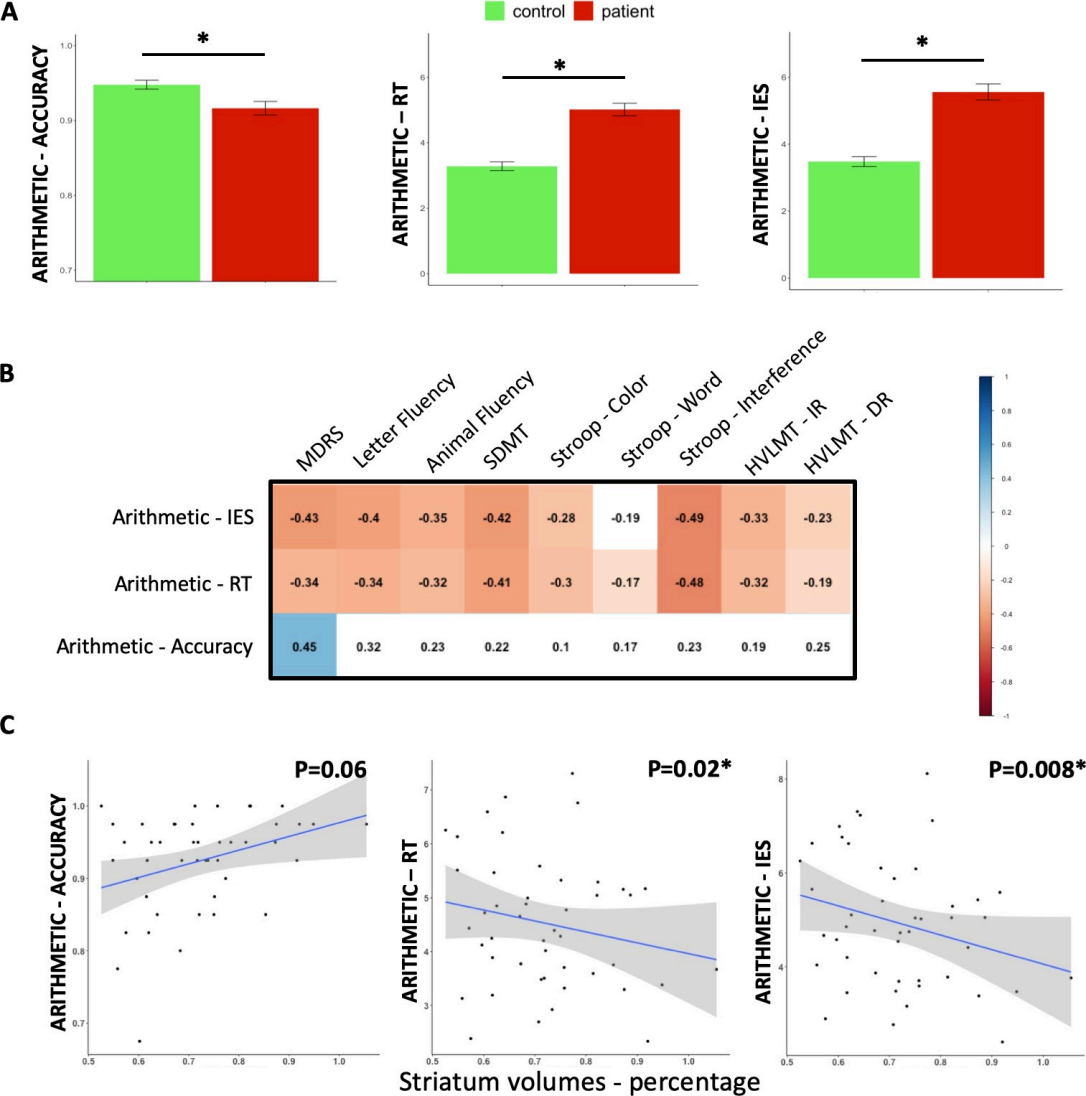
Baseline results. A–Discriminant validity at baseline: significant differences between groups are represented by a star (p<0.05). B—Pearson correlations between arithmetic scores and cognitive measures (Bonferroni correction, p-values * 8) in HD patients. Blue colour indicates significant positive correlations and red colour significant negative correlation. White colour indicates non-significant correlation. C–Association between striatal volumes and accuracy, response time and IES in the arithmetic task (across the two operations) in HD patients. MDRS Mattis Dementia Rating Scale, SDMT symbol digit modalities test, HVLT Hopkins Verbal Memory Test, IR immediate recall, DR delayed recall.

Similarly, in DAT (Fig 2A), controls were more accurate and faster than HD patients (respectively: estimates β = -0.04, SE = 0.011, P = 0.001; β = 1.83, SE = 0.24, P<0.001). Controls also had a lower arithmetic IES than HD patients (β = -2.23, SE = 0.30, P<0.0001). Slower RT and higher IES were associated with TFC (respectively: β = -0.56, SE = 0.18, P = 0.002; β = 0.03, SE = 0.009, P = 0.0008) but not with TMS (all Ps >0.05). In HD patients, Arithmetic RT and Arithmetic IES correlated with most cognitive measures, while Arithmetic accuracy only correlated with MDRS (Fig 2B).

Striatal volumes were smaller in HD patients than in controls (P<0.0001). Slower Arithmetic RT and higher Arithmetic IES were associated with increased striatal atrophy in the HD group (respectively: β = -2.93, SE = 1.25, P = 0.02; β = -3.81, SE = 1.36, P = 0.008; Fig 2C), as well as with TMS, SDMT, animal fluency, letter fluency, Stroop-Color, Stroop-Word and immediate recall of the HVLMT (S2 and S3 Figs). In contrast, there was no significant association between striatal volume and Arithmetic accuracy, Stroop-Interference, MDRS, or delayed recall of the HVLMT (all Ps > 0.05).

On average, participants were retested at M1 after 30.10 days ± 14.22 (range 14–115). As reported in Table 2, both controls and HD patients performed better at M1 compared to M0 in Arithmetic RT and Arithmetic IES with a main effect of time, but no interaction between group and time. Test-retest reliability ranged from r = 0.64 to r = 0.91 for Arithmetic RT and Arithmetic IES in both groups. Test-retest reliability for accuracy was r = 0.69 in HD patients and r = 0.28 in the control group due to a ceiling effect in the latter (mean accuracy was approximately 0.95 in controls). Results on separate operations (S1 Table) are similar to those obtained with global scores across the two operations.

**Table 2:** Baseline and Month 1 comparisons on the DAT.

DAT scores	Groups	Month 0	Month 1	Group	Time	Group * Time	r	p
Arithmetic—IES	Controls	3.48 ± 1.11	3.31 ± 1.06	<0.0001	0.0008	0.22	0.91	<0.0001
Arithmetic—IES	HD patients	5.56 ± 2.10	5.23 ± 1.57				0.64	<0.0001
Arithmetic—Accuracy	Controls	94.78 ± 4.56	95.88 ± 5.16	<0.0001	0.3	0.28	0.28	0.036
Arithmetic—Accuracy	HD patients	91.62 ± 7.92	91.55 ± 7.06				0.69	<0.0001
Arithmetic—RT	Controls	3.28 ± 1.005	3.15 ± 0.942	<0.0001	0.003	0.24	0.89	<0.0001
Arithmetic—RT	HD patients	5.02 ± 1.67	4.74 ± 1.32				0.79	<0.0001

Means and standard deviations at Month 0 and Month 1 at the Digitalized Arithmetic Task in each group and statistical results on main effects of group, time and their interaction. On the right, the Pearson’s correlation coefficients between the two tests are shown with their p-values.

### Longitudinal analysis (M1-M12)

In the longitudinal subset (S3 Table), HD patients and controls did not differ in age, sex, or years of education (all Ps>0.05). In HD patients, mean TFC score decreased from baseline to M12 (M0: 10.49 ± 1.70, range: 7–13; M12: 9.87 ± 2.25, range: 5–13; β = -0.72, SE = 0.17, P<0.0001). Mean TMS remained stable over time (β = 1.74, SE = 1.28, P = 0.18; baseline score: 30.30 ± 15.10 (range: 1–60); M12 score: 31.33 ± 15.65 (range:1–67)).

**Fig 3:**
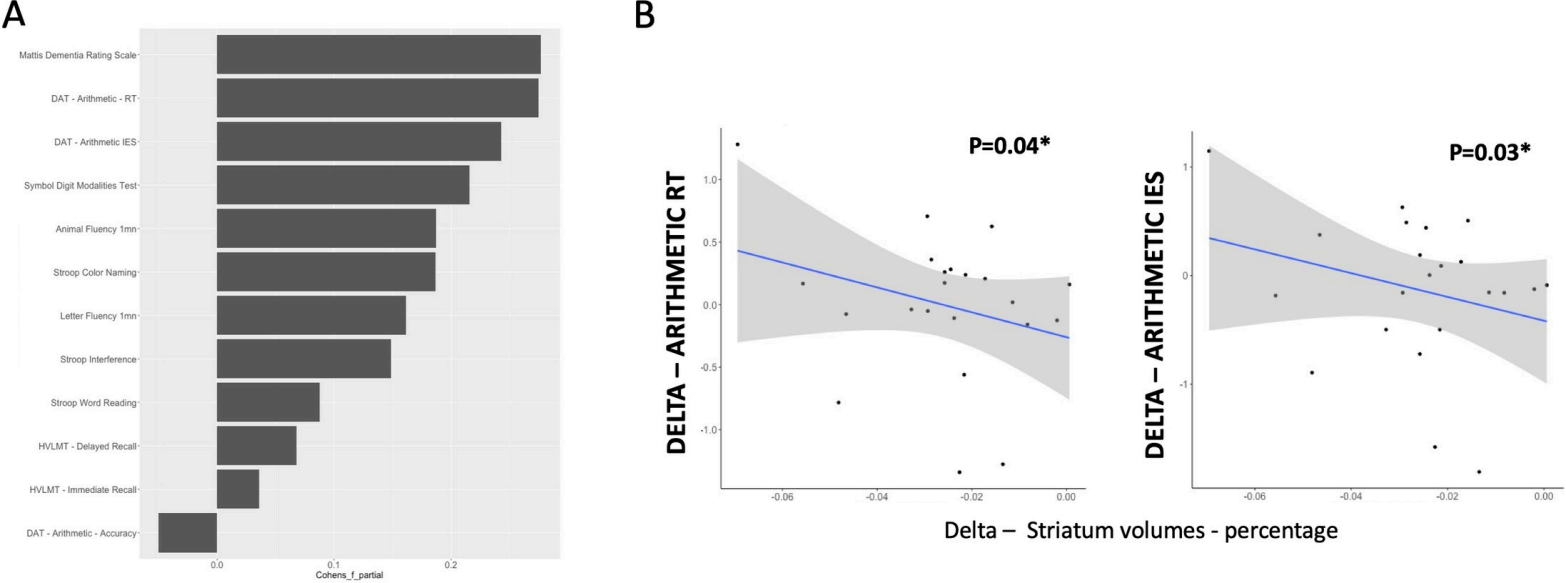
Longitudinal results. A–Longitudinal Cohen’s f effect sizes for each cognitive test between month 1 and month 12 evaluations. B–Association between striatal change and response time and IES changes between Month 1 and Month 12 in HD patients. IES Inverse Efficiency Score, HVLT Hopkins Verbal Memory Test, DAT Digitalized Arithmetic Test, RT Response Time, IES Inverse Efficiency Score.

Performance in controls remained stable up to M12 for each of the cognitive tests, except for the Stroop Word Reading test, in which they improved (Table 3). In contrast, HD patients’ performance declined in MDRS, SDMT, and DAT (Arithmetic RT and Arithmetic IES). RT performance decline was observed in both the subtraction and multiplication operations (S4 Table). However, performance remained stable in HD patients for verbal fluency, Stroop tests, HVLMT, and Arithmetic accuracy. Longitudinal effect sizes for Arithmetic RT and Arithmetic IES from M1 to M12 (Cohen’s f effect sizes: 0.27 and 0.24, respectively) were similar to those obtained with MDRS (Cohen’s f effect size: 0.28) and higher than those obtained with the other neuropsychological tests (all Cohen’s f effect sizes <0.21) (Fig 3A).

**Table 3:** Results of model comparisons testing the main effect of time, interaction of time and group (Controls and HD patients) and post-hoc comparisons testing the M1-M12 change in performance in Controls and in HD patients.

Cognitive tasks	Main effects and interaction			Controls					HD patients				
	Group	Time	Group * Time	Month 1	Month 12	estimate	SE	p values	Month 1	Month 12	estimate	SE	p values
Arithmetic—IES	0.005*	**0.07**	0.27	3.32 ± 0.92	3.41 ± 0.82	0.084	0.18	0.64	5.27 ± 1.63	5.66 ± 2.20	0.343	0.152	0.027*
Arithmetic—Accuracy	0.47	0.82	0.41	96.18 ± 5.51	95.59 ± 4.13	-0.006	0.01	0.5	90.94 ± 7.63	91.20 ± 7.89	0.004	0.008	0.64
Arithmetic—RT	0.0007*	**0.05**	0.18	3.17 ± 0.82	3.25 ± 0.72	0.061	0.14	0.67	4.72 ± 1.25	5.06 ± 1.63	0.303	0.119	0.01*
MDRS	**0.09**	**0.07**	0.14	142.0 ± 1.94	141.8 ± 2.26	-0.222	0.919	0.81	133.5 ± 8.97	131.2 ± 9.59	-1.984	0.776	0.01*
Animal Fluency	0.0005*	0.19	0.35	24.53 ± 6.15	24.32 ± 5.30	-0.192	0.79	0.81	16.14 ± 5.68	14.74 ± 4.72	-1.152	0.667	**0.09**
Letter Fluency	0.0003*	0.07	0.89	49.09 ± 9.18	47.61 ± 11.06	-1.608	1.475	0.28	31.97 ± 11.3	29.88 ± 10.3	-1.863	1.246	0.14
SDMT	<0.0001*	**0.06**	0.46	53.41 ± 9.26	52.68 ± 10.09	-0.62	0.833	0.46	31.63 ± 9.20	29.62 ± 10.24	-1.419	0.706	0.047*
Stroop Word Reading	<0.0001*	0.27	0.03*	102.0 ± 12.00	106.5 ± 14.19	4.405	2.067	0.04*	70.26 ± 18.28	67.91 ± 14.59	-1.417	1.745	0.42
Stroop Color Naming	<0.0001*	0.78	**0.05**	81.93 ± 12.94	83.52 ± 12.59	1.651	1.501	0.27	52.76 ± 14.88	49.75 ± 12.35	-2.194	1.268	**0.09**
Stroop Interference	<0.0001*	0.94	**0.09**	47.97 ± 9.88	48.97 ± 10.74	1.178	1.11	0.29	29.44 ± 9.19	27.58 ± 8.40	-1.289	0.938	0.17
HVLMT—IR	0.0002*	0.33	0.59	29.15 ± 3.19	29.82 ± 4.09	0.681	0.692	0.33	20.56 ± 5.26	20.52 ± 5.92	-0.195	0.585	0.74
HVLMT—DR	<0.0001*	0.83	0.55	10.85 ± 1.26	10.97 ± 1.45	0.094	0.374	0.8	6.819 ± 3.21	6.566 ± 3.04	-0.196	0.316	0.54

Means (and standard deviations) are presented for each test at each time of evaluation. IES Inverse Efficiency Score, RT Response Time, MDRS Mattis Dementia Rating Scale, SDMT Symbol Digit Modalities Test, HVLMT Hopkins Verbal Learning Memory Test, IR Immediate Recall, DR Delayed Recall.

Striatal volume regression analysis revealed an interaction between groups (controls, HD patients) and time (M1, M12) (X^2^(1) = 18.46, P<0.001). A progressive reduction in striatal volume was observed in HD patients between M1 and M12 (β = 0.025, SE = 0.004, P<0.0001) but not in controls (P>0.05). Slower Arithmetic RT, increased Arithmetic IES and decline in TFC between M1 and M12 were associated with decrease in striatal volumes (respectively: β = -17.03, SE = 7.52, P = 0.037; β = -18.51, SE = 7.59, P = 0.027; β = 7.44, SE = 3.61, P = 0.047) (Fig 3B and S3 Fig). In contrast, striatal volume decrease was associated neither with the Arithmetic accuracy nor with TMS or cognitive decline in any of the paper and pencil tasks (all Ps >0.05, S4 Fig).

## Discussion

We report the validity of the Digitalized Arithmetic Task (DAT) and its sensitivity to capture cognitive decline in HD patients at stages 1 and 2 of the disease. This study was conducted in the framework of the European multi-centric Repair-HD study in four centers and three languages (French, English, and German). In approximately 4 minutes, DAT not only allowed for discrimination between groups (controls vs. HD patients), but also detected a decline in cognitive performance over one year in a relatively small cohort of HD patients (N = 48). Effect sizes were similar to those obtained in both the general cognitive assessment (MDRS) and executive (SDMT) paper and pencil tasks. In contrast with the UHDRS cognitive assessment, MDRS and HLVMT, longitudinal decline in DAT performance was associated with striatal atrophy, a major pathological hallmark of HD [10], and was not impacted by motor decline. Our findings strongly suggest DAT as a cognitive endpoint candidate for future clinical trials.

Our study complements the recently adopted multidomain approach to increase the sensitivity of impairment monitoring in HD. Some authors have used a composite score combining performances from several clinical and cognitive tests [11,32] rather than using several related tests, each testing a single domain entity [9]. This strategy aims to reduce the failure of cognitive tests to show a systematic decline across studies such as in [33,34]. In this present study, we show that the development of tasks simultaneously assessing two cognitive domains might also be a pertinent strategy to obtain a powerful tool for monitoring cognition in HD. Designed from the theoretical framework for the striatum’s role in cognition, DAT assesses fronto-subcortical language and executive deficits through the respective verification of multiplication and subtraction [21]. These cognitive domains appear to be sensitive not only for assessing cognitive status, but also for tracking cognition in HD patients [9,10,16,35]. Comparison of effect sizes indicated that our task was more sensitive to one-year decline in this cohort than the widely used cognitive component of the UHDRS. Moreover, the association of striatal atrophy with arithmetic performance (digitalized or paper and pencil versions) in early-HD and pre-HD patients [22], as well as with its metabolic activity for the paper and pencil version in early HD [18], reinforces the use of this strategy to improve cognitive assessment.

When developing a cognitive test, careful consideration must be given to its psychometric validation. Following the recommendations of Mestre and collaborators [5], clinical studies are currently aiming to validate cognitive batteries used in HD monitoring such as the HD-CAB [11]. Consistent with this need, we addressed this question in this study. The DAT showed excellent psychometric properties. At baseline, DAT measures (response time and Inverse Efficiency Score, IES) were highly associated with global cognitive impairment, Stroop tests, SDMT, verbal fluency, MDRS, HTLMT, and striatal volume in HD patients. The test-retest reliability rated good to excellent for response time and IES, in whichever language it was administered (French, English, or German). As expected, we found a retest effect with improvement in DAT’s response time between M0 and M1 in controls and HD patients without significant change in accuracy due to a ceiling effect. However, conducting two evaluations over a short period of time and then using the second evaluation as a baseline reduced the practice effect and discrepancies between patients who have not been previously evaluated and patients who are already familiar with the tasks (Stout et al., 2014; Schramm et al., 2015) [11,12]. We strongly recommend this strategy for future trials, in order to increase the reliability of longitudinal assessments.

Whereas cognitive decline progresses slowly in HD, DAT has been able to show a decline over one year in a relatively small cohort of patients. Among classic cognitive tests included in our study, only the Mattis Dementia Rating Scale (MDRS) and the Symbol Digit Modalities Test (SDMT) were found to show a significant decline in this time frame. Surprisingly, despite extensive use in the 1980s and 1990s (see for example: [36,37]), MDRS (which assesses global cognitive function) is currently no longer really used with HD patients, presumably because of its difficult translation and its duration (on average 30 minutes in advanced stages). However, our study shows an annual decline with a slope of 1.98 +/- 0.78 point with MDRS, contrasting with our results in a follow-up study of 22 patients over 2 to 4 years, where the annual decline was not significant [38]. This might rely on the larger number of participants, and the reduction of the retest effect thanks to the subsequent baseline assessments (M0-M1) [38].

Considering the decline in other classic paper and pencil tasks, only SDMT demonstrated a decline in our cohort in accordance with previous large and multicentric longitudinal studies, suggesting that speed processing measures are the most reliable indices of disease progression [9,10]. We suggest that measures combining both speed processing and accuracy (such as SDMT, Stroop tests, verbal fluency, and DAT) are more efficient for longitudinal follow-up than tasks assessing only accuracy or processing speed. By using precise response time recording, Arithmetic response time could be more efficient in showing a decline over one year when compared to traditional time-dependent tasks such as Stroop tests or SDMT because of its digitalization, limiting examiner bias. Furthermore, it eliminates linguistic differences, which might hamper getting positive results in small cohorts. For example, the letters chosen for fluency tasks are different between countries, as well as the number of syllables when denominating colors. In addition to accuracy, response time allows for a single comprehensive measure, the Arithmetic IES [28], combining both accuracy and response time. This measure acknowledges that considering accuracy and response time separately may not capture the whole picture in patients’ cognitive decline. IES is widely used to measure cognition in healthy subjects as well as in patients with neurological injury [39,40] because it enhances the validity of cognitive measures reflecting “the average energy consumed by the system” [41]. When responding to a cognitive task, participants can be fast at the expense of accuracy or accurate at the expense of speed, yielding to a so-called “speed accuracy trade-off” [29]. Here, some HD patients increased their response time between month 1 and month 12, but 23% decreased their accuracy, while 42% increased the latter. Such different patterns might show a change in the participant’s strategy or the need for longer information processing time to respond correctly, and not only a cognitive decline preventing the patient from responding to the task correctly. Thus, because patients’ behavior may evolve over time, the IES appears necessary to measure disease progression in long-lasting studies or in studies combing various stages of HD patients. Furthermore, it has a large longitudinal effect size and is associated with striatal volume.

This study shows that the use of DAT, a fast, cognitive, digitalized task developed for use in HD, has the potential to improve clinical practice by increasing standardization, reliability, and efficiency of cognitive assessment, as well as by automating the scoring process in longitudinal follow-up studies. By combining two key cognitive functions affected by HD, as well as the response time and accuracy through the comprehensive IES metric, DAT is sensitive to disease progression over one-year; DAT is likely to be useful in other neurodegenerative diseases. The DAT could be used to monitor cognitive status in HD patients included in clinical trials as well as in clinical follow-up. The DAT is available free of charge upon request, by writing to the corresponding author (ACBL). Thus, it also could be used in low-resource settings to assess and monitor cognitive status, when clinicians and researchers cannot access expensive assessments such as neuropsychological tests and an MRI scan. Future studies should assess whether the promising DAT has the capacity to be completed at home by HD patients, as this could reduce the financial cost and inconvenience for patients and families requiring frequent hospital and clinic visits. In addition, a longitudinal follow-up study of gene-carrier individuals on this task is important to assess whether the cognitive measures identified in this present study can be used as markers even for individuals far from predicted clinical symptom onset. This is especially crucial during the current developmental era of disease-modifying treatments for this pathology. Finally, the design of the present study did not allow for the comparison between the digitalized and paper and pencil versions of the arithmetic task used previously [18]. To investigate the potential superiority of the digitalized version (which now provides a response time), a future study should be conducted in a new sample of participants with healthy controls and HD patients.

## Supporting information

S1 Fig Correlations between TFC, TMS and classical paper and pencil tasks significant negative correlations are represented by the red color (p<0.05, Bonferroni correction for multiple comparisons). TFC Total Functional Capacity, TMS Total Motor Score, SDMT Symbol Digit Modalities Test, HVLMT Hopkins Verbal Learning Memory Test, IR Immediate recall, DR Delayed Recall, MDRS Mattis Dementia Rating Scale.
(DOCX)

S2 Fig Association between striatal volumes (in percentage) and DAT results at baseline (Month 0) in HD patients RT Response Time. Arithmetic IES, Arithmetic accuracy and Arithmetic RT are the score across the two conditions (subtraction and multiplication).
(DOCX)

S3 Fig Association between striatal volumes (in percentage) and cognitive performances at baseline (Month 0) in HD patients. TMS Total Motor Score, MDRS Mattis Dementia Rating Scale, SDMT Symbol Digit Modalities Test, HVLMT Hopkins Verbal Learning Memory Test, RI Immediate recall, RD Delayed Recall.
(DOCX)

S4 Fig Association between change (delta M12-M1) in striatal volumes (in percentage) and change in clinical scores and DAT performances (delta M12-M1) over one-year in HD patients. TFC Total Functional capacity, TMS Total Motor Score, RT Response Time, IES Inverse Efficiency Score Arithmetic IES, Arithmetic Accuracy and Arithmetic RT are the score across the two conditions (subtraction and multiplication).
(DOCX)

S5 Fig Association between change (delta M12-M1) in striatal volumes (in percentage) and change in cognitive scores (delta M12-M1) over one-year in HD patients. MDRS Mattis Dementia Rating Scale, SDMT Symbol Digit Modalities Test, HVLMT Hopkins Verbal Learning Memory Test, RI Immediate recall, RD Delayed Recall.
(DOCX)

S1 Table Means and standard deviations at Month 0 and Month 1 at the Digitalized Arithmetic Task according to the type of operation in each group and statistical results on main effects of group, Time and their interaction. On the right, the Pearson’s correlation coefficients between the two tests are shown with their p-values.
(DOCX)

S2 Table Mean (and standard deviation) of neuropsychological performances at baseline (Month 0) and statistical results on group differences. (DOCX)

S3 Table Participant demographics in the longitudinal subset. Unless otherwise specified, values are means ± standard deviations.
(DOCX)

S4 Table Results of models comparisons in DAT according to the operation testing the main effect of time, interaction of time and group (Controls and HD patients) and post-hoc comparisons testing the M1-M12 change in performance in controls and in HD patients. Means (and standard deviations) are presented for each test at each time of evaluation.
(DOCX)
