# Supplementary material for: Cognitive decline in Huntington’s disease in the Digitalized Arithmetic Task (DAT)
Source: PLoS One. 2021 Aug 23;16(8):e0253064. doi: 10.1371/journal.pone.0253064 (PMC8382187; doi:10.1371/journal.pone.0253064)
Supplement: S1 Fig — TFC Total Functional Capacity, TMS Total Motor Score, SDMT Symbol Digit Modalities Test, HVLMT Hopkins Verbal Learning Memory Test, IR Immediate recall, DR Delayed Recall, MDRS Mattis Dementia Rating Scale. (DOCX) [file pone.0253064.s001.docx]

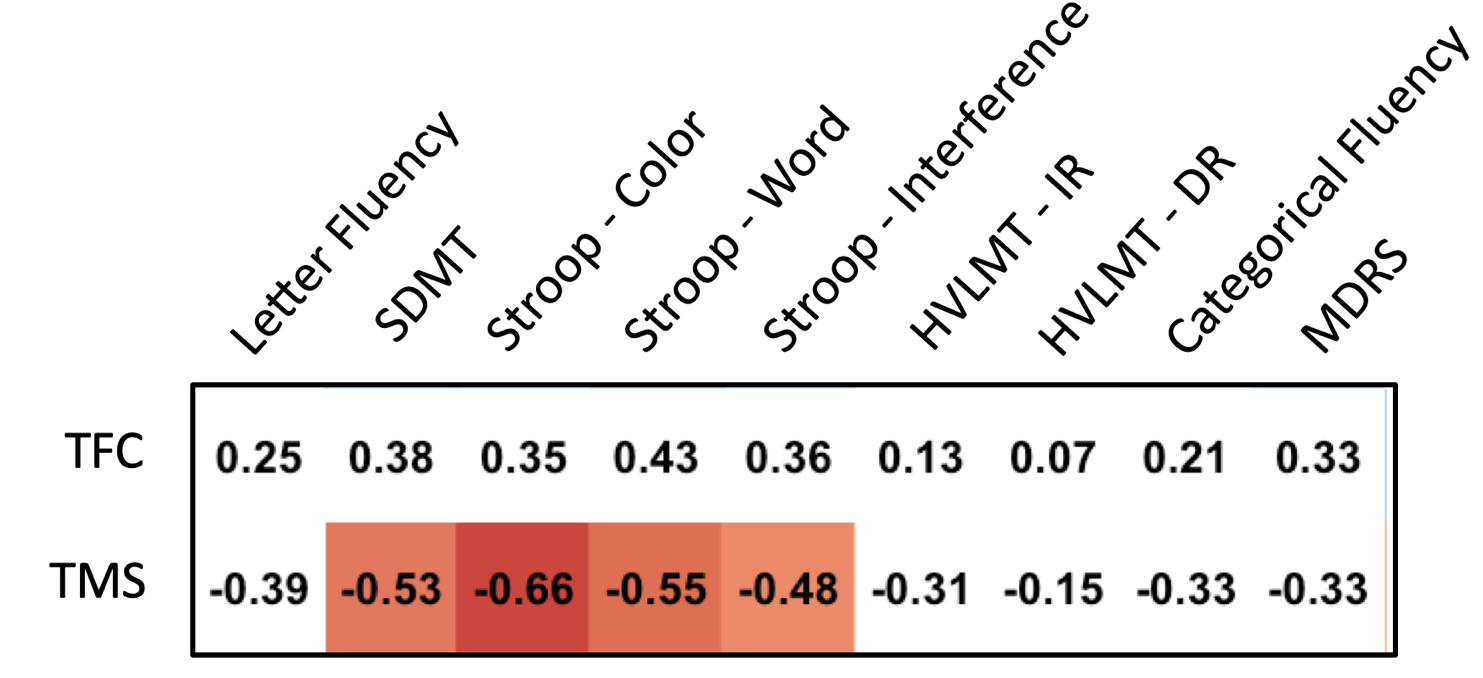


**Supplementary Figure 1**: Correlations between TFC, TMS and classical paper and pencil tasks

Significant negative correlations are represented by the red color (p<0.05, Bonferroni correction for multiple comparisons).

TFC Total Functional Capacity, TMS Total Motor Score, SDMT Symbol Digit Modalities Test, HVLMT Hopkins Verbal Learning Memory Test, IR Immediate recall, DR Delayed Recall, MDRS Mattis Dementia Rating Scale.
