# Supplementary material for: Cognitive decline in Huntington’s disease in the Digitalized Arithmetic Task (DAT)
Source: PLoS One. 2021 Aug 23;16(8):e0253064. doi: 10.1371/journal.pone.0253064 (PMC8382187; doi:10.1371/journal.pone.0253064)
Supplement: S2 Fig — Arithmetic IES, Arithmetic accuracy and Arithmetic RT are the score across the two conditions (subtraction and multiplication). (DOCX) [file pone.0253064.s002.docx]

**
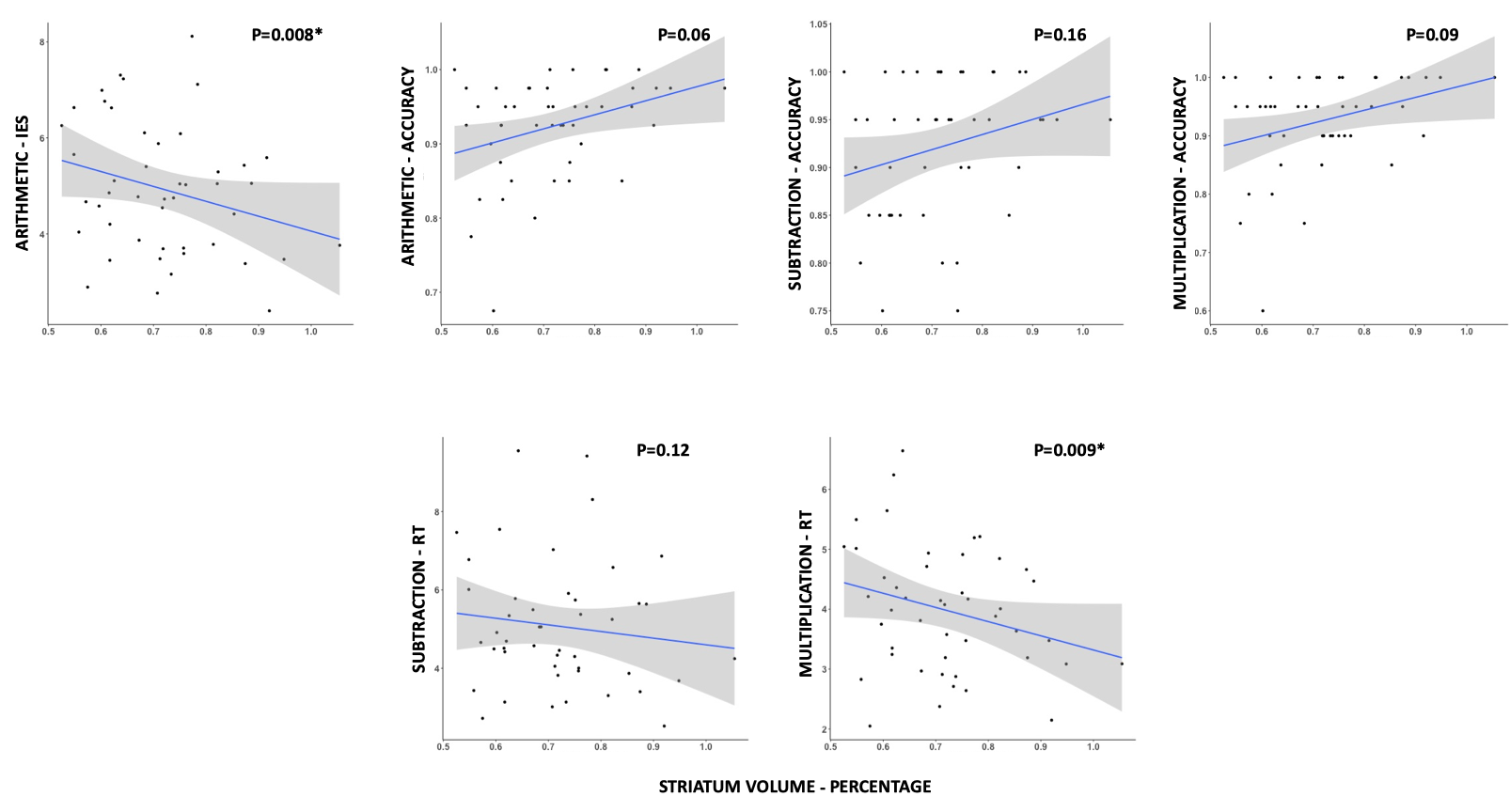
**

**Supplementary Figure 2:** Association between striatal volumes (in percentage) and DAT results at baseline (Month 0) in HD patients

RT Response Time

Arithmetic IES, Arithmetic Accuracy and Arithmetic RT are the score across the two conditions (subtraction and multiplication)
