# Supplementary material for: Cognitive decline in Huntington’s disease in the Digitalized Arithmetic Task (DAT)
Source: PLoS One. 2021 Aug 23;16(8):e0253064. doi: 10.1371/journal.pone.0253064 (PMC8382187; doi:10.1371/journal.pone.0253064)
Supplement: S3 Fig — TMS Total Motor Score, MDRS Mattis Dementia Rating Scale, SDMT Symbol Digit Modalities Test, HVLMT Hopkins Verbal Learning Memory Test, RI Immediate recall, RD Delayed Recall. (DOCX) [file pone.0253064.s003.docx]

**
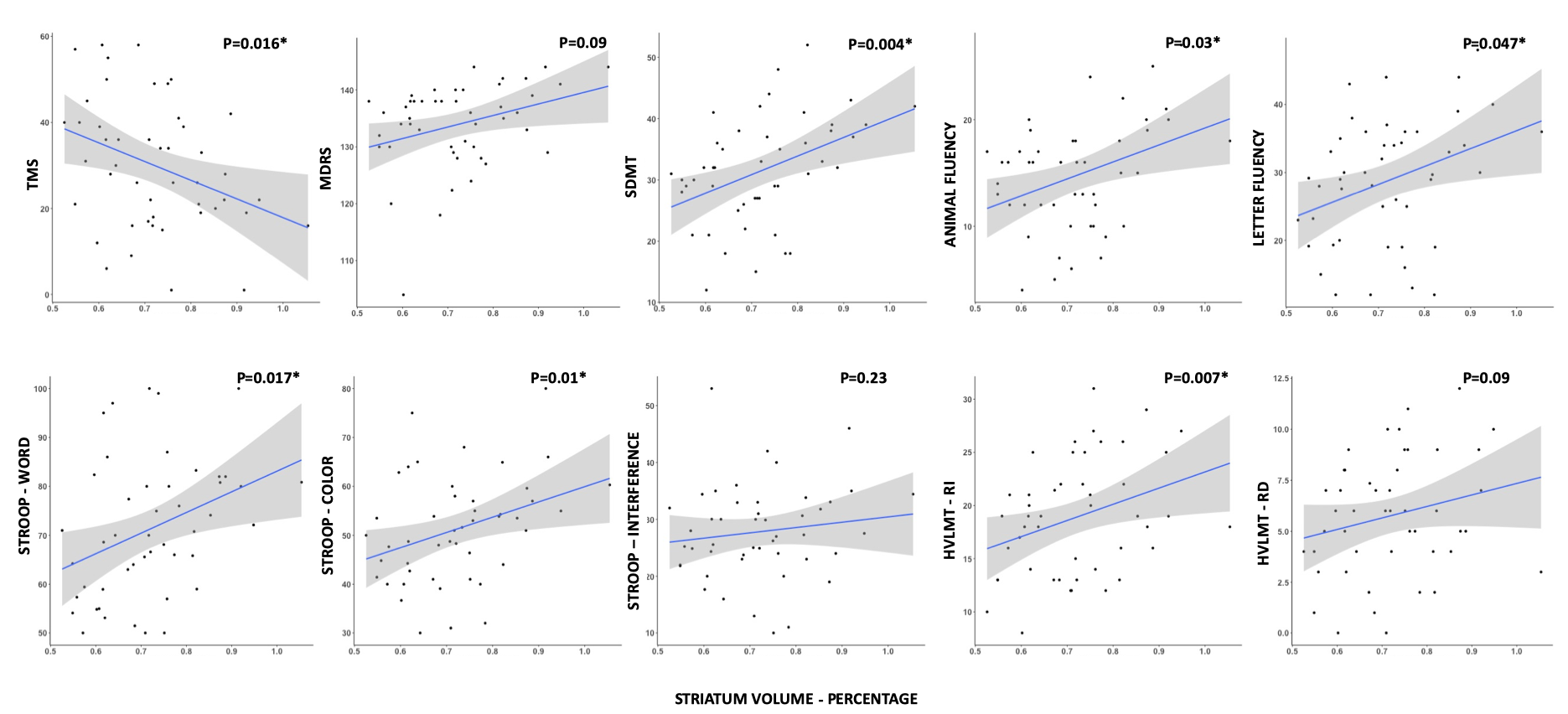
**

**Supplementary Figure 3:** Association between striatal volumes (in percentage) and cognitive performances at baseline (Month 0) in HD patients

TMS Total Motor Score, MDRS Mattis Dementia Rating Scale, SDMT Symbol Digit Modalities Test, HVLMT Hopkins Verbal Learning Memory Test, RI Immediate recall, RD Delayed Recall
