# Supplementary material for: Cognitive decline in Huntington’s disease in the Digitalized Arithmetic Task (DAT)
Source: PLoS One. 2021 Aug 23;16(8):e0253064. doi: 10.1371/journal.pone.0253064 (PMC8382187; doi:10.1371/journal.pone.0253064)
Supplement: S4 Fig — TFC Total Functional capacity, TMS Total Motor Score, RT Response Time, IES Inverse Efficiency Score Arithmetic IES, Arithmetic Accuracy and Arithmetic RT are the score across the two conditions (subtraction and multiplication). (DOCX) [file pone.0253064.s004.docx]

**
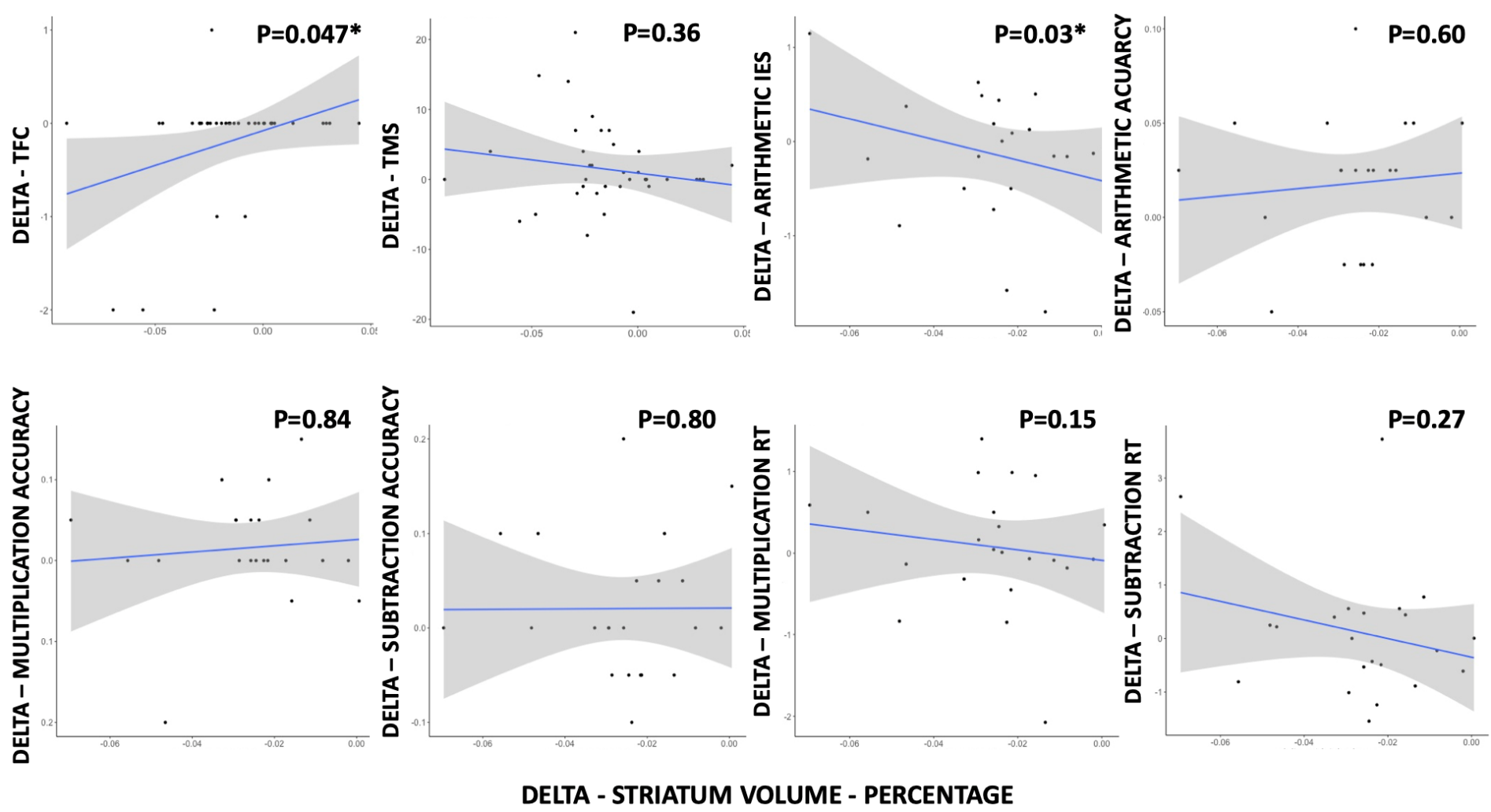
**

**Supplementary Figure 4:** Association between change (delta M12-M1) in striatal volumes (in percentage) and change in clinical scores and DAT performances (delta M12-M1) over one-year in HD patients

TFC Total Functional capacity, TMS Total Motor Score, RT Response Time, IES Inverse Efficiency Score

Arithmetic IES, Arithmetic Accuracy and Arithmetic RT are the score across the two conditions (subtraction and multiplication)
