# Supplementary material for: Cognitive decline in Huntington’s disease in the Digitalized Arithmetic Task (DAT)
Source: PLoS One. 2021 Aug 23;16(8):e0253064. doi: 10.1371/journal.pone.0253064 (PMC8382187; doi:10.1371/journal.pone.0253064)
Supplement: S5 Fig — MDRS Mattis Dementia Rating Scale, SDMT Symbol Digit Modalities Test, HVLMT Hopkins Verbal Learning Memory Test, RI Immediate recall, RD Delayed Recall. (DOCX) [file pone.0253064.s005.docx]

**
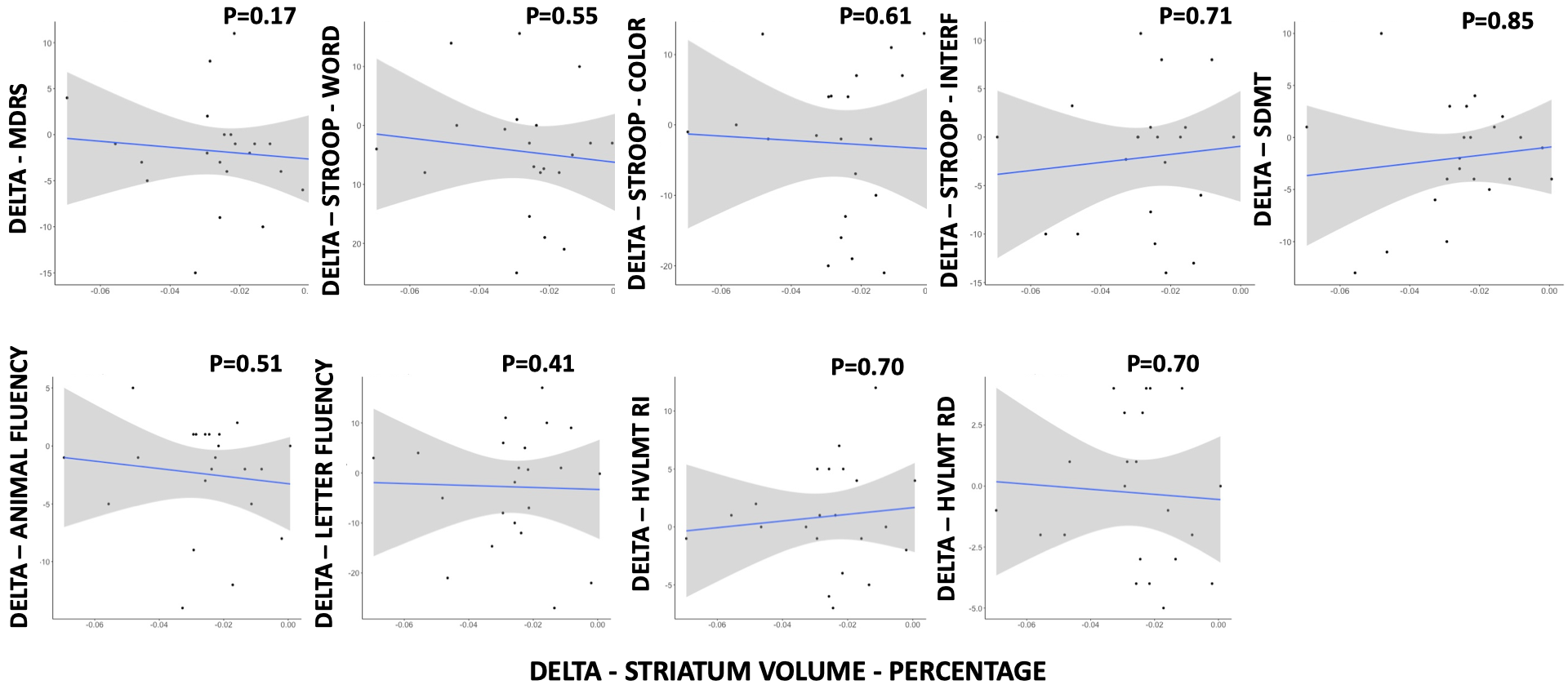
**

**Supplementary Figure 5:** Association between change (delta M12-M1) in striatal volumes (in percentage) and change in cognitive scores (delta M12-M1) over one-year in HD patients

MDRS Mattis Dementia Rating Scale, SDMT Symbol Digit Modalities Test, HVLMT Hopkins Verbal Learning Memory Test, RI Immediate recall, RD Delayed Recall
