# Supplementary material for: Cognitive decline in Huntington’s disease in the Digitalized Arithmetic Task (DAT)
Source: PLoS One. 2021 Aug 23;16(8):e0253064. doi: 10.1371/journal.pone.0253064 (PMC8382187; doi:10.1371/journal.pone.0253064)
Supplement: S1 Table — On the right, the Pearson’s correlation coefficients between the two tests are shown with their p-values. (DOCX) [file pone.0253064.s006.docx]

**Supplementary Table 1:** Means and standard deviations at Month 0 and Month 1 at the Digitalized Arithmetic Task according to the type of operation in each group and statistical results on main effects of group, Time and their interaction. On the right, the Pearson’s correlation coefficients between the two tests are shown with their p-values.

| **DAT scores** | **Groups** | **Month 0** | **Month 1** | **Group** | **Time** | **Group * Time** | **r** | **p** |
| --- | --- | --- | --- | --- | --- | --- | --- | --- |
| Multiplication - Accuracy | Controls | 95.00 ± 5.09 | 95.79 ± 5.89 | 0.003 | 0.62 | 0.43 | 0.16 | 0.22 |
| Multiplication - Accuracy | HD patients | 92.66 ± 8.49 | 92.43 ± 8.23 |  |  |  | 0.64 | <0.0001 |
| Multiplication - RT | Controls | 3.72 ± 1.42 | 3.39 ± 1.31 | <0.0001 | 0.002 | 0.66 | 0.82 | <0.0001 |
| Multiplication - RT | HD patients | 5.42 ± 2.17 | 4.98 ± 1.73 |  |  |  | 0.63 | <0.0001 |
| Subtraction - Accuracy | Controls | 94.56 ± 6.36 | 95.96 ± 6.51 | 0.0001 | 0.31 | 0.38 | 0.4 | 0.002 |
| Subtraction - Accuracy | HD patients | 90.58 ± 10.23 | 90.66 ± 8.69 |  |  |  | 0.52 | <0.0001 |
| Subtraction - RT | Controls | 3.92 ± 1.75 | 3.63 ± 1.23 | <0.0001 | 0.048 | 0.86 | 0.83 | <0.0001 |
| Subtraction - RT | HD patients | 5.89 ± 2.88 | 5.68 ± 2.38 |  |  |  | 0.77 | <0.0001 |

Accuracy for subtraction was lower than for multiplication (main effect of operation; X^2^(2)=14.44, P<0.001, without an interaction between group and operation (X^2^(1)=1.46, P=0.23)). RTs are slower in subtraction than in multiplication (main effect of operation; X^2^(1)=37.97, P<0.001, without interaction between group and operation (X^2^(1)=0.31, P=0.58)).

RT Response Time
