# Supplementary material for: Cognitive decline in Huntington’s disease in the Digitalized Arithmetic Task (DAT)
Source: PLoS One. 2021 Aug 23;16(8):e0253064. doi: 10.1371/journal.pone.0253064 (PMC8382187; doi:10.1371/journal.pone.0253064)
Supplement: S2 Table — (DOCX) [file pone.0253064.s007.docx]

**Supplementary Table 2**: Mean (and standard deviation) of neuropsychological performances at baseline (Month 0) and statistical results on group differences.

| **Neuropsychological tasks** | **Controls** | **HD patients** | **β** | **SE** | **p** |
| --- | --- | --- | --- | --- | --- |
| MDRS | 142.0 ± 2.27 | 132.8 ± 7.81 | -9.13 | 1.07 | <0.0001 |
| Letter Fluency | 43.45 ± 9.19 | 28.74 ± 9.29 | -14.7 | 1.63 | <0.0001 |
| Animal Fluency | 22.44 ± 6.26 | 15.05 ± 4.96 | -7.38 | 0.98 | <0.0001 |
| SDMT | 52.73 ± 10.00 | 32.05 ± 9.65 | -20.68 | 1.72 | <0.0001 |
| Stroop Color | 79.91 ± 11.92 | 50.85 ± 11.39 | -29.06 | 2.03 | <0.0001 |
| Stroop Word | 101.90 ± 14.05 | 69.73 ± 14.70 | -32.17 | 2.52 | <0.0001 |
| Stroop Interference | 47.96 ± 11.26 | 27.41 ± 8.49 | -20.55 | 1.71 | <0.0001 |
| HVLMT Immediate_Recall | 28.30 ± 3.59 | 19.61 ± 6.06 | -8.68 | 0.9 | <0.0001 |
| HVLMT Delayed_Recall | 10.32 ± 1.69 | 6.01 ± 3.09 | -4.31 | 0.45 | <0.0001 |

MDRS Mattis Dementia Rating Scale; SDMT Symbol Digit Modality Test, HVLMT Hopkins Verbal Learning Memory Test; IR Immediate recall; DR Delayed recall;

Decrease of performance in HD patients compared to controls are expressed by negative estimates.
