# Supplementary material for: Cognitive decline in Huntington’s disease in the Digitalized Arithmetic Task (DAT)
Source: PLoS One. 2021 Aug 23;16(8):e0253064. doi: 10.1371/journal.pone.0253064 (PMC8382187; doi:10.1371/journal.pone.0253064)
Supplement: S3 Table — Unless otherwise specified, values are means ± standard deviations. (DOCX) [file pone.0253064.s008.docx]

**Supplementary Table 3:** Participant Demographics in the longitudinal subset.

Unless otherwise specified, values are means ± standard deviations.

|  | Controls | HD patients |
| --- | --- | --- |
| Number | 34 | 48 |
| (Cardiff/Créteil/Manchester/Muenster) | (3/22/2/7) | (8/26/4/10) |
| Laterality | 1A/29R/4L | 2A/44R/2L |
| Sex | 15F/19M | 21F/27M |
| Age (years) | 51.22 ± 10.81 | 53.65 ± 11.51 |
| [range] | [26.17- 68.42] | [23.21- 72.98] |
| Education (years) | 14.44 ± 3.60 | 13.98 ± 3.19 |
| [range] | [9- 24] | [9- 20] |
| TFC (M0) | 13.00 ± 0.00 | 10.81 ± 1.57 |
| [range] | [13- 13] | [7- 13] |
| CAG repeat | - | 43.35 ± 4.13 |
| [range] | - | [38 - 62] |
| Age of onset (years) | - | 50.00 ± 11.06 |
| [range] | - | [21 - 66] |
| Disease duration (years) | - | 4.85 ± 4.12 |
| [range] | - | [0.29 - 19.20] |
| Disease burden score | - | 389.02 ± 105.88 |
| [range] |  | [ 134.60 - 675.35] |
