# Supplementary material for: Cognitive decline in Huntington’s disease in the Digitalized Arithmetic Task (DAT)
Source: PLoS One. 2021 Aug 23;16(8):e0253064. doi: 10.1371/journal.pone.0253064 (PMC8382187; doi:10.1371/journal.pone.0253064)
Supplement: S4 Table — Means (and standard deviations) are presented for each test at each time of evaluation. (DOCX) [file pone.0253064.s009.docx]

| **Cognitive tasks** | **Main effects and interaction** | | | **Controls** | | | | | **HD patients** | | | | |
| --- | --- | --- | --- | --- | --- | --- | --- | --- | --- | --- | --- | --- | --- |
|  | **Group** | **Time** | **Group * Time** | **Month 1** | **Month 12** | **estimate** | **SE** | **p values** | **Month 1** | **Month 12** | **estimate** | **SE** | **p values** |
| Multiplication - Accuracy | 0.24 | 0.13 | 0.97 | 95.74 ± 6.64 | 96.91 ± 4.93 | -0.011 | 0.011 | 0.34 | 91.67 ± 8.83 | 92.81 ± 7.92 | -0.011 | 0.009 | 0.238 |
| Multiplication - RT | **0.06** | 0.16 | 0.14 | 2.99 ± 1.03 | 2.99 ± 0.73 | 0.007 | 0.14 | 0.96 | 4.25 ± 1.11 | 4.57 ± 1.36 | -0.263 | 0.119 | **0.03*** |
| Subtraction - Accuracy | 0.98 | 0.17 | 0.31 | 96.62 ± 6.60 | 94.26 ± 6.41 | 0.024 | 0.015 | 0.12 | 90.21 ± 9.78 | 89.58 ± 11.38 | 0.004 | 0.013 | 0.77 |
| Subtraction - RT | **0.006*** | 0.05 | 0.26 | 3.50 ± 1.05 | 3.63 ± 0.87 | -0.11 | 0.21 | 0.6 | 5.52 ± 1.96 | 5.99 ± 2.68 | -0.418 | 0.178 | **0.02*** |

**Supplementary Table 4:** Results of models comparisons in DAT according to the operation testing the main effect of time, interaction of time and group (Controls and HD patients) and post-hoc comparisons testing the M1-M12 change in performance in Controls and in HD patients. Means (and standard deviations) are presented for each test at each time of evaluation
